# Identification of Differentially Expressed Proteins from *Leishmania amazonensis* Associated with the Loss of Virulence of the Parasites

**DOI:** 10.1371/journal.pntd.0002764

**Published:** 2014-04-03

**Authors:** Rubens D. M. Magalhães, Mariana C. Duarte, Eliciane C. Mattos, Vivian T. Martins, Paula S. Lage, Miguel A. Chávez-Fumagalli, Daniela P. Lage, Daniel Menezes-Souza, Wiliam C. B. Régis, Maria J. Manso Alves, Manuel Soto, Carlos A. P. Tavares, Ronaldo A. P. Nagen, Eduardo A. F. Coelho

**Affiliations:** 1 Departamento de Bioquímica e Imunologia, Instituto de Ciências Biológicas, Universidade Federal de Minas Gerais, Belo Horizonte, Minas Gerais, Brazil; 2 Programa de Pós-Graduação em Ciências Saúde: Infectologia e Medicina Tropical, Faculdade de Medicina, Universidade Federal de Minas Gerais, Belo Horizonte, Minas Gerais, Brazil; 3 Departamento de Bioquímica, Instituto de Química, Universidade de São Paulo, São Paulo, São Paulo, Brazil; 4 Departamento de Patologia Clínica, COLTEC, Universidade Federal de Minas Gerais, Belo Horizonte, Minas Gerais, Brazil; 5 Departamento de Parasitologia, Instituto de Ciências Biológicas, Universidade Federal de Minas Gerais, Belo Horizonte, Minas Gerais, Brazil; 6 Departamento de Bioquímica, PUC Minas, Belo Horizonte, Minas Gerais, Brazil; 7 Centro de Biología Molecular Severo Ochoa (CSIC-UAM), Departamento de Biología Molecular, Universidad Autónoma de Madrid, Madrid, Spain; Yale School of Public Health, United States of America

## Abstract

**Background:**

The present study analyzed whether or not the *in vitro* cultivation for long periods of time of pre-isolated *Leishmania amazonensis* from lesions of chronically infected BALB/c mice was able to interfere in the parasites' infectivity using *in vivo* and *in vitro* experiments. In addition, the proteins that presented a significant decrease or increase in their protein expression content were identified applying a proteomic approach.

**Methodology/Principal Findings:**

Parasites were cultured *in vitro* for 150 days. Aliquots were collected on the day 0 of culture (R0), as well as after ten (R10; 50 days of culture), twenty (R20; 100 days of culture), and thirty (R30; 150 days of culture) passages, and were used to analyze the parasites' *in vitro* and *in vivo* infectivity, as well as to perform the proteomic approach. Approximately 837, 967, 935, and 872 spots were found in 2-DE gels prepared from R0, R10, R20, and R30 samples, respectively. A total of 37 spots presented a significant decrease in their intensity of expression, whereas a significant increase in protein content during cultivation could be observed for 19 proteins (both cases >2.0 folds). Some of these identified proteins can be described, such as diagnosis and/or vaccine candidates, while others are involved in the infectivity of *Leishmania*. It is interesting to note that six proteins, considered hypothetical in *Leishmania*, showed a significant decrease in their expression and were also identified.

**Conclusions/Significance:**

The present study contributes to the understanding that the cultivation of parasites over long periods of time may well be related to the possible loss of infectivity of *L. amazonensis*. The identified proteins that presented a significant decrease in their expression during cultivation, including the hypothetical, may also be related to this loss of parasites' infectivity, and applied in future studies, including vaccine candidates and/or immunotherapeutic targets against leishmaniasis.

## Author Summary

*Leishmania amazonensis* can induce a diversity of clinical manifestations in mammal hosts, including tegumentary and visceral leishmaniasis. The present study evaluated the variation of infectivity of *L. amazonensis*, which was pre-isolated from lesions of chronically infected mice and *in vitro* cultured for 150 days, in turn connecting these results with the profile of parasite protein expression using a proteomic approach. Parasites were recovered after the first passage, as well as after 50, 100, and 150 days of axenic cultures, and were subsequently evaluated. A total of 37 proteins presented a significant decrease, whereas 19 proteins presented a significant increase in their protein expression content in the assays (both cases >2.0 fold). Some of the identified proteins have been reported in prior literature, including diagnosis and/or vaccine candidates for leishmaniasis, while others proved to be involved in the infectivity of *Leishmania*. It is interesting to note that proteins related to the parasites' metabolism were also the majority of the proteins identified in the old cultures of *L. amazonensis*, suggesting a possible relation between the metabolic state of parasites and their possible loss of infectivity. In conclusion, the proteins identified in this study represent a contribution to the discovery of new vaccine candidates and/or immunotherapeutic targets against leishmaniasis.

## Introduction

Leishmaniasis consists of a wide range of diseases present in 98 countries worldwide, where approximately 1.6 million cases occur each year, with an estimated 40,000 deaths [1]. Many geographic regions are endemic for multiple *Leishmania* species, which is the case in Brazil, where the disease is caused by at least six different species of *Leishmania*. Among them, *Leishmania amazonensis* presents a particular importance, as it is one of the main species capable of causing human disease with a broad spectrum of clinical manifestations, ranging from cutaneous to visceral leishmaniasis [2], [3]. In one study, it was also observed that BALB/c mice experimentally infected with *L. amazonensis* developed visceralization of the parasites in different organs, such as the brain, liver, spleen, and bone marrow, characterizing a diagnosis of murine visceral leishmaniasis [4].

It has been postulated that the *in vitro* maintenance of parasites by cultivation over long periods of time may well diminish their ability to differentiate into amastigote forms [5]. In fact, long-term axenic cultures were one of the first empirical approaches to efficiently identify parasite virulence genes, which later led to the experimental development of attenuated strains [6]. Similarly, the long-term *in vitro* growth of drug-resistant parasites was suggested to mediate the loss of resistance phenotype [7]. It is well-known that parasites can regulate their gene expression, mainly at the post-transcriptional level; however, little is known about the biological mechanisms and the protein expression involved in this process [8]. In this context, the identification of proteins involved either in the infectivity of parasites in the mammal hosts, or in their maintenance in axenic cultures, should be considered relevant.

The proteomic study applied to evaluate the protein expression patterns in *Leishmania* offers the possibility of assigning potential functions for proteins, including those previously identified by genomics as hypothetical, which should be evaluated, such as vaccine candidates, diagnostic markers, and/or immunotherapeutic targets. Several studies have been published evaluating the stage-specific expression and differentiation profiles of proteins in different *Leishmania* species [9], [10], [11], [12], [13], [14]. In addition, the discovery of new proteins through proteomics has been recommended as one of the main research priorities for further development and improvement of leishmaniasis vaccines [15].

In this context, the identification of proteins involved in parasites' infectivity should be considered important, given that they could be used in immunological applications to prevent the disease. In the present study, a proteomic approach, based on two-dimensional electrophoresis (2-DE) and mass spectrometry, was carried out to analyze the variation of protein expression profiles in stationary promastigotes of *L. amazonensis*, which were pre-isolated from lesions of chronically infected BALB/c mice and maintained in axenic cultures over a long period of time. The proteins that presented significant variations in their levels during the *in vitro* cultivation were identified in an attempt to select new vaccine candidates and/or immunotherapeutic targets against leishmaniasis. The results showed several known, as well as six hypothetical, *L. amazonensis* proteins, some of which are well-known proteins involved in the infectivity of *Leishmania*, while others are described through the metabolic functions of the parasites.

## Materials and Methods

### Ethics statement

Experiments were performed in compliance with the National Guidelines of the Institutional Animal Care and Use Committee for the Ethical Handling of Research Animals (CEUA) from the Federal University of Minas Gerais (UFMG) (Law number 11.794, 2008), which approved this study on April 25, 2012, under protocol number 092/2012.

### Mice and parasites

Female BALB/c mice (8 weeks of age) were obtained from the breeding facilities of the Department of Biochemistry and Immunology, Institute of Biological Sciences, UFMG, and were maintained under specific pathogen-free conditions. *L. amazonensis* (IFLA/BR/1967/PH-8) parasites were grown at 24°C in complete Schneider's medium , supplemented with 20% heat-inactivated fetal bovine serum (FBS), 20 mM L-glutamine, 200 U/ml penicillin, 100 µg/ml streptomycin, and 50 µg/ml gentamicin, at pH 7.4. The amastigote-like cells were obtained as described in [16].

### *In vivo* infection

BALB/c mice (n = 8) were infected subcutaneously in their hind footpad with 1×10^6^ stationary promastigotes of *L. amazonensis*. The course of the disease was monitored at weekly intervals by measuring footpad thickness with a metric caliper, and expressed as the increase in thickness of the infected footpad compared to the non-infected footpad. At week 8 post-infection, animals were sacrificed and their infected footpads, spleen, and liver were harvested for parasite quantification by a limiting-dilution assay [17]. To evaluate the *in vivo* infectivity of parasites in the different collected passages, R0 and R30 samples were used to infect BALB/c mice (n = 8, each group). The infection schedule and the parasitological analyses were the same as described above.

### Preparation of the parasites for proteomics

Parasites were collected from infected footpads of the animals (8 weeks after infection) and purified to perform the proteomic approach. For this, parasites recovered from lesions were homogenized and immediately washed in Schneider's medium, which was supplemented with 10% FBS and 1% penicillin G/streptomycin sulfate solution, and subsequently cultured in complete Schneider's medium. Passages of *in vitro* cultures were performed every five days, until the thirtieth passage (150 days after). Aliquots were collected on day 0 of culture (R0, first passage), as well as 50 (R10), 100 (R20) and 150 (R30) days after the beginning of the cultures, and quantified for the experiments.

### Evaluation of *in vitro* infectivity

Aliquots containing parasites of R0, R10, R20, and R30 passages were centrifuged for 10 min and 5,000× *g*, at 4°C. The supernatant was removed, and the pellet containing the parasites was washed 3 times with sterile PBS. Murine macrophages collected from BALB/c mice were plated on round glass coverslips within the wells of a 24-well culture plate, at a concentration of 5×10^5^ cells per coverslip in RPMI 1640 medium, which was supplemented with 20% FBS, 2 mM L-glutamine, 200 U/mL penicillin G, and 100 µg/mL streptomycin sulfate, at pH 7.4. After 2 h of incubation at 37°C in 5% CO_2_, stationary promastigotes of *L. amazonensis* were quantified and added to the wells (1×10^6^ and 5×10^6^, for a ratio of 1∶2 or 1∶10 macrophage per parasites, respectively). The cultures were incubated for 24 h at 37°C in 5% CO_2_. Next, the cells were washed and stained to determine the percentages of infected macrophages and the number of intra-macrophage amastigotes by counting 200 cells in triplicate [18]. An optical microscopy was also used to check the stationary profile of all *in vitro* cultures, and a prior titration curve was performed to determine the best time of infection for the macrophages (data not shown).

### Preparation of total extract of *Leishmania*

The total extraction of proteins of *L. amazonensis* was performed following a technical protocol [19]. Briefly, 2×10^8^ stationary promastigotes were dissolved in a DeStreak rehydratation solution, containing phosphatases (5 mM NaF, 2 mM Na_3_VO_4_, and 50 mM Na β-glycerophosphate) and proteases (Protease Inhibitor Cocktail; plus 1 mM PMSF) inhibitors. After homogenization, samples were disrupted by sonication in an ice bath for 15 min by applying a continuous pulse and centrifuged at 20,000× *g* for 7 min, at 4°C. The supernatant was collected, and the protein concentration was estimated using the Bradford method [20]. Aliquots were immediately frozen at −80°C, until use. For each passage, cellular material was extracted from the parasites harvested from two different animals, and two independent culture bottles of each animal were grown separately, totaling four individual samples.

### Isoelectric focusing

The isoelectric focalization (IEF) was performed using the Ettan IPGphor3 system. For the first-dimension electrophoresis, 650 µg of total extracts were added to a volume of 250 µL with a rehydration solution containing a DeStreak rehydratation solution in 1% immobilized pH gradient buffer (IPG-buffer, pH 4–7). Next, samples were applied to IPG strips (13 cm, pH 4–7; GE Healthcare) for passive rehydration for 18 h at room temperature. After gel rehydration, IEF was performed at 1,000 V for 800 V/h; 8,000 V for 11,500 V/h; holding at 8,000 V for 7,500 V/h.

### SDS-PAGE

After IEF, each strip was incubated for 15 min with 1% dithiothreitol (DTT) in the equilibrium buffer [75 mM Tris-HCl buffer, pH 8.8; 6 M urea, 39% (v/v) glycerol, and 2% (w/v) SDS], followed by a second incubation step for 15 min in 2.5% iodoacetamide diluted in equilibrium buffer. IPG strips were washed with milli-Q water, transferred to a 12% polyacrilamide, and sealed with an agarose solution (0.5% agarose in running buffer, containing 25 mM Tris, 192 mM glycine, and 0.1% SDS, pH 8.3). The protein standard was purchased from BioRad (pre-stained SDS-PAGE broad range). Electrophoresis was performed using a SE 600 ruby standard dual cooled vertical unit system connected to a MultiTemp III cooling bath. Proteins were separated at 30 mA/gel.

### Protein digestion, peptide extraction, and spot handling

The 2-DE gels were stained with colloidal Coomassie Brilliant Blue G-250, following a defined technical procedure [21]. For image analysis, 16 stained gels were scanned using an ImageScanner III. Analyses were carried out using ImageMaster 2D Platinum 7.0 software. This software identifies spots on a gel image (300 dpi) by comparing the number of pixels in the background image to the number of pixels that make up the image of the spot itself. The spots present in the images are differentiated from other gels by determining the spot's position through the manual insertion of image markers. The parameters used for spot detection included: minimal area of 5 pixels, with a smooth factor of 4 and a saliency of 80. The reference gel (higher number of spots) was used to match corresponding protein spots within different gels. The intensity volume of individual spots was normalized by the total intensity volume (value of the intensity volumes obtained from all spots in the same 2-DE gel) so as to remain relatively independent of variations due to protein loading and staining, performed by considering the total volume of all spots in the images. All of the spots selected by software were checked manually. The statistical test of analysis of variance (One-way ANOVA) was performed at a 1% statistical significance level (*P*<0.01) to determine the mean values of spot intensity for each passage (R0, R10, R20, and R30) in an attempt to determine the significant changes among the passages. Additionally, this study applied a cut-off of at least 2-fold of the core value of intensity of all spots selected by the program, which were the same in each passage. The obtained fold value was the number obtained by the ratio between the higher and lower core values of each spot's passage. Spots that presented significant variations within the passages were manually excised and destained with a solution containing 50% methanol and 2.5% acetic acid. The proteins were reduced in 10 mM DTT and alkylated using 50 mM iodoacetamide. Limited protein enzymatic digestion was performed with 0.4 or 0.8 µg of trypsin for larger spots. Excess protease was removed and replaced by 25 mM ammonium bicarbonate. Digestion was performed at 37°C for 18 h. Peptide extraction was performed twice for 15 min, using 30 µL of a solution containing 50% acetonitrile and 5% formic acid. The digested samples were dried using a speed-vac.

### Protein identification and database search

The identification of proteins was performed at the Mass Spectrometry Laboratory of the Brazilian Biosciences National Laboratory (LNBio, CNPEM/ABTLuS, Campinas, São Paulo, Brazil). This procedure was conducted using an ESI-Quad-TOF apparatus attached to a UPLC system. The mass spectra were processed by the Protein Lynx V 2.1 program and analyzed by the MASCOT MS/MS Ion Search program (http://www.matrixscience.com). The following parameters were used for this analysis: enzyme, trypsin; allowing of up to 1 missed cleavage; fixed modification, carbamidomethyl (C); variable modification, oxidation (M); peptide tolerance, ±0.1 Da; MS/MS tolerance, ±0.1 Da; and a peptide charge of 1+, 2+, and 3+. The database *Leishmania* (dated June 2012) was used for protein identification, the records of which can be found in the NCBI concerning *Leishmania spp.* (49,496 sequences; 30,861,888 residues). All data regarding the proteins evaluated in the present study were harvested from NCBI, UniProt, and Gene Ontology databases.

### Immunoblotting 2-DE analysis

To validate the proteins identified in this study, such as the significant decrease or increase in their expression content after cultivation, Western blot experiments and 2-DE gel quantitation were performed. Whole cell extracts of stationary promastigotes and amastigotes-like forms of *L. amazonensis* were separated electrophoretically from R0 and R30 passages and transferred onto cellulose membranes (Schleicher & Schull, Dassel, Germany) by semi-dry blotting for 2 h at 400 mA. Membranes were blocked in 5% (w/v) low-fat dried milk diluted in TBS plus 0.05% Tween 20 for 16 h at 4°C. Next, the membranes were washed 3 times with a solution containing TBS and 0.05% Tween 20 (TBS-T, 10 min each) and were pre-incubated with anti-α-tubulin (1∶1,000 dilution), anti-HSP83 (1∶1,000 dilution), anti-GRP78 (1∶2,000 dilution), or anti-paraflagellar rod protein 1D (1∶2,000 dilution) antibodies for 2 h at room temperature. After, membranes were washed 6 times with TBS-T (10 min each) and incubated with a peroxidase-conjugated anti-rabbit IgG secondary antibody (1∶40,000 dilution) for 2 h at room temperature. After having been washed 7 times with TBS-T (10 min each), the reaction was processed using ECL™ Western Blotting Detection Reagent and ImageQuant LAS4000 equipment. The Ponceau S staining of each membrane was used as a loading control (data not shown). The band intensity of each protein was quantified by Image J software. The normalized values were obtained in the comparison between R0 and R30 of each parasite stage. The experiments were performed in triplicate, and the Student's t-test (*P*<0.05) was employed in the statistical analyses.

### Statistical analysis

The statistical analysis of the *in vitro* and *in vivo* infectivity experiments was performed using the GraphPad Prism software (version 5.0 for Windows). The differences were evaluated by one-way ANOVA analysis, followed by the Bonferroni' test. Differences were considered significant when *P*<0.05. Statistical analyses evaluating the intensity and variation of the protein expression profile in the 2-DE gels and immunoblotting were also performed, as described above. The data are representative of three independent experiments, performed in triplicate, which presented similar results.

## Results

### Evaluation of *in vivo* and *in vitro* infectivity

**Figure 1:**
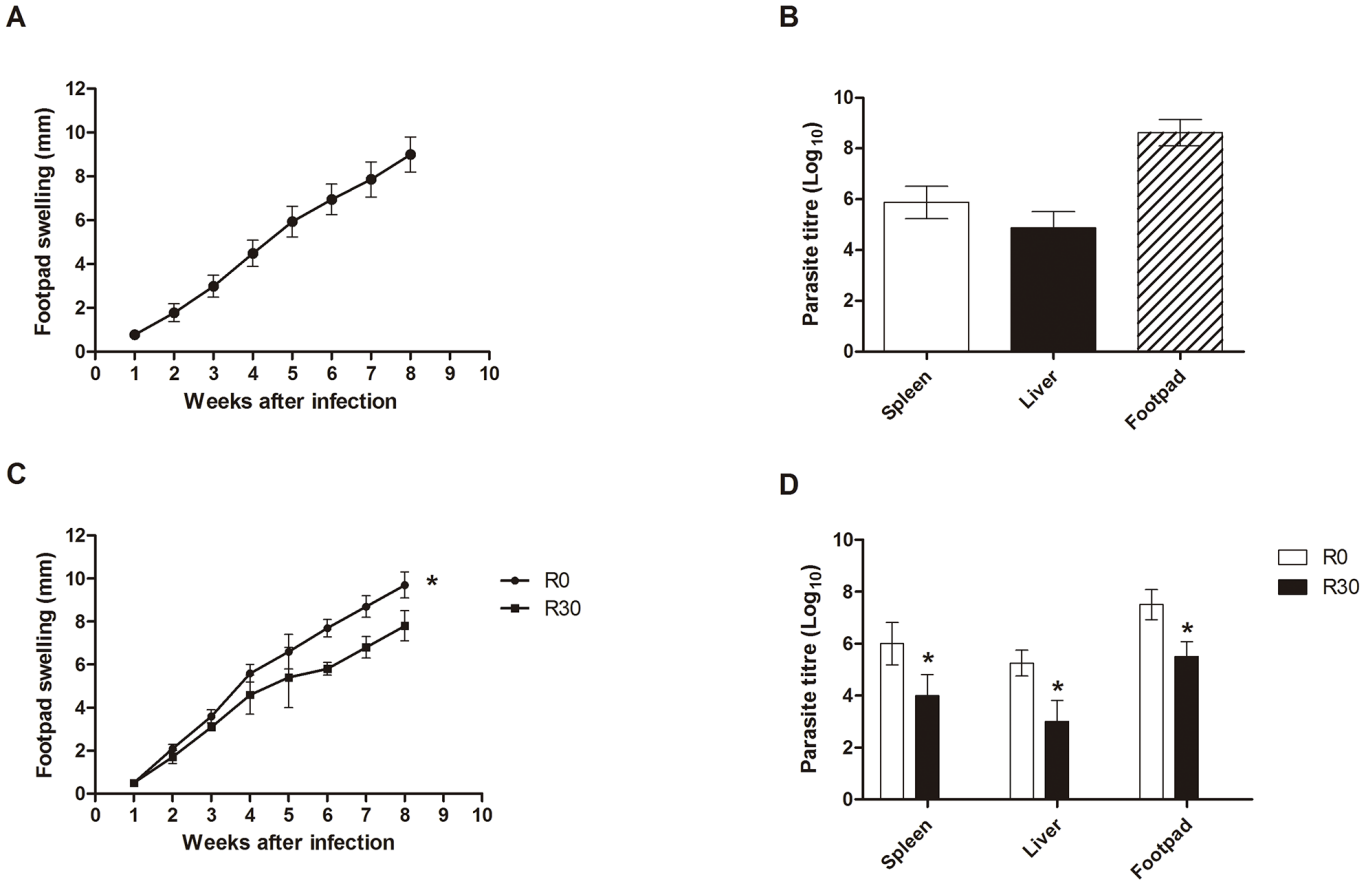
Infection of BALB/c mice. Mice (n = 8) were infected subcutaneously with 1×10^6^ stationary promastigotes of *Leishmania amazonensis*. Lesion development in the infected footpads was monitored weekly, up to 8 weeks after infection. Mean ± standard deviation (SD) are shown in (A). Parasite load in the infected footpads, spleen, and liver was analyzed in all animals (B). Other mice (n = 8, per group) were subcutaneously infected with 1×10^6^ stationary promastigotes of *L. amazonensis* obtained from R0 or R30 passages, and the lesion development was monitored up to 8 weeks after infection. Mean ± SD of the groups are shown (C). The parasite load in the infected footpads, spleen, and liver was also evaluated in these groups (D). The experiments were repeated three times, and presented similar results. *Significant difference between the R0 and R30 groups (*P*<0.05).

BALB/c mice (n = 8) subcutaneously infected with *L. amazonensis* were monitored for 8 weeks by measuring the footpad thickness, given that the footpad swelling was similar in all evaluated animals (Figure 1A). The number of parasites recovered in the infected footpads, spleen, and liver of the infected animals was evaluated, and the results showed values of 8.6±0.5, 5.9±0.6, and 4.9±0.6 log, respectively (Figure 1B). In this context and due to the high homogeneity of infections in the mice, represented by similar values of footpad swelling and parasite loads, the parasites were recovered from lesions and used in the axenic cultures to perform the proteomic analyses of this study.

To evaluate the variation of the *in vivo* infectivity between the different passages of *L. amazonensis*, stationary promastigotes obtained from R0 and R30 samples were used to infect BALB/c mice (n = 8 per group, with 1×10^6^ stationary promastigotes injected in each mouse). Animals infected with R30, as compared to the animals infected with R0, presented a significantly lower edema in the infected footpads at 8 weeks after infection (Figure 1C). The lower lesion size observed in the R30 group, when compared to the values obtained in the R0 group, was related to the lower parasite load observed when evaluating the infected footpads, spleen, and liver of these animals (Figure 1D).

For the evaluation of the *in vitro* infectivity, stationary promastigotes recovered in all passages (R0, R10, R20, and R30) were quantified and employed in the experiments. It could be observed that by using 2 parasites to infect 1 macrophage, parasites obtained from the R0 passage presented an infection average of 65.1±1.5% and a number of amastigotes per macrophage of 2.2±0.1. By contrast, using the R30 sample, the infection average was 14.9±2.3% and the number of amastigotes per macrophage was 0.5±0.1. When 10 parasites were used to infect 1 macrophage, the infection average of the R0 group was 96.9±2.6% and the number of amastigotes per macrophage was 7.4±0.4. On the other hand, using parasites from the R30 group, the infection average was 59.5±2.2% and the number of amastigotes per macrophage was 3.8±0.4 (Table 1).

**Table 1:** Evaluation of *in vitro* infection.

Ratio	Percentage of infected macrophages			
	R0	R10	R20	R30
1∶2	65.1±1.5	37.8±3.2	19.2±3.6	14.9±2.3
1∶10	96.9±2.6	82.8±1.4	64.1±2.5	59.5±2.2

Ratio	Number of amastigotes per macrophage			
	R0	R10	R20	R30
1∶2	2.2±0.1	1.6±0.1	0.7±0.2	0.5±0.1
1∶10	7.4±0.4	5.2±0.8	4.3±0.2	3.8±0.4

Murine macrophages (5×10^5^ cells) were infected with stationary promastigotes of *L. amazonensis* (1×10^6^ and 5×10^6^, by a ratio of 1∶2 or 1∶10 macrophage per parasites, respectively) and the cultures were incubated for 24 h at 37°C, 5% CO_2_. Next, free parasites were removed and the percentage of infected cells and the number of amastigotes per macrophage in each passage (R0, R10, R20, and R30) were analyzed by counting 200 cells in triplicate. Mean ± SD is shown. Data shown are representative of three separate experiments, performed in triplicate, which presented similar results.

### Analyses of protein expression in *Leishmania amazonensis*

**Figure 2:**
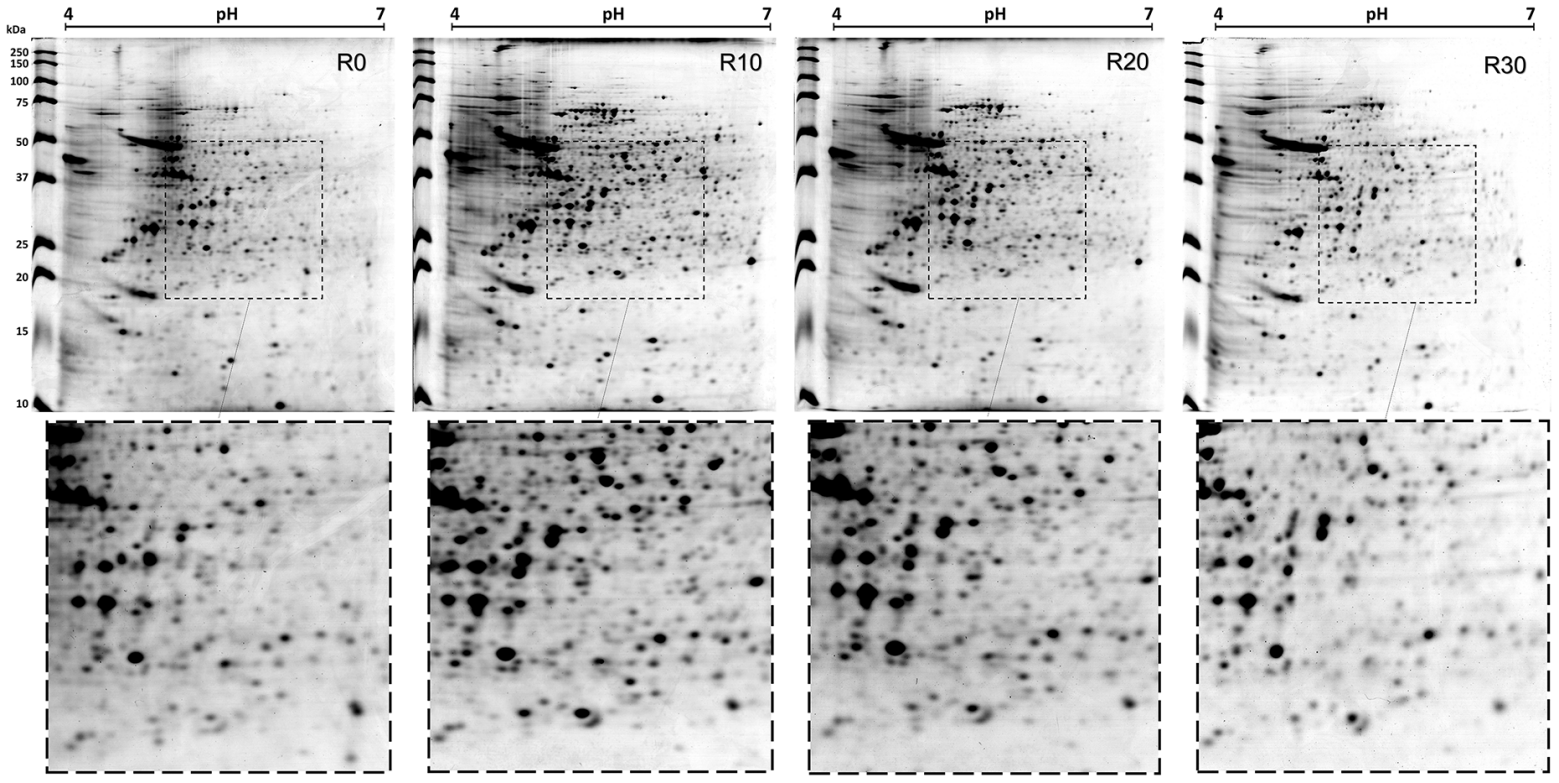
Two-dimensional profiles of cultures from *Leishmania amazonensis*. The 2-DE gels were obtained after the separation of stationary promastigotes extracts (R0, R10, R20, and R30 passages; 650 µg of each extract) by 2-DE (first dimension: IEF pH range 4–7; second dimension: 12% SDS-PAGE) and staining with colloidal Coomassie Brilliant Blue G-250. The gel fragments in the lower portion of the figures represent evaluated amplifications (see within the dotted lines). 2-DE gels of each passage were derived from four independent protein preparations of each passage. One representative preparation of each sample is showed in this study.

Electrofocusing was performed using 13 cm pH 4–7 IPG strips after having investigated the best strip to isolate the total extracts. Strips of 13 cm were chosen because they provide a better range of separation of proteins by their *pI* without the gels becoming difficult to handling. Two ranges of pH were evaluated: 3–10 and 4–7. This study opted for a narrower pH range, given that most of the identified spots were located in this region. Although some spots located outside the pH 4–7 have been missed, the most spots were obtained within of this range due a better separation. After 2-DE gels had been applied, approximately 837 spots were found in the R0 sample, while 967, 935, and 872 spots were identified in the R10, R20, and R30 samples, respectively. Figure 2 is representative of the gels obtained in each condition. The 2-DE profiles and the number of observed spots in the different passages were reproducible in terms of both the total number of protein spots and their relative positions and intensities in four 2-DE gels performed for each passage (data not shown). After 2-DE analysis, 315 spots, which presented a significant variation in their intensities, were selected for identification by mass spectrometry. From all these spots, 258 were identified as proteins, and 164 unique proteins were identified. Of these, 58 proteins showed that the intensity of their corresponding spots either increased (19 spots) or decreased (37 spots) during the passages from R0 to R30, always maintaining a 2-fold minimal variation. It is also important to report that, upon performing the *in vitro* infection experiments, a stabilization of the infectivity could be observed between the R20 and R30 samples (Table 1).

### Identification of proteins of interest

Among the 37 proteins that presented a significant decrease in their content during the axenic passages, six were hypothetical, while another 31 were known proteins, like described in Table 2 [22]–[52]. Some of these proteins present biological functions described in prior literature, such as tryparedoxin peroxidase [25], metallo-peptidases [29], heat shock protein HSP70 [33], and protein disulfide isomerase [48], all of which are involved with the parasites' infectivity. Possible targets for therapeutic interventions, such as S-adenosylmethionine synthetase [47]; proteins identified as diagnosis candidates, such as acidic ribosomal protein P2; and vaccine candidates, such as the eukaryotic initiation factor 4A [34] and thiol-dependent reductase 1 [52], were also identified. Proteins involved in the flagellum motility in *Leishmania*, such as a small myristoylated protein [22], and others related to metabolic functions, such as aldehyde dehydrogenase [28], were also identified. Evaluating the proteins that presented a significant increase in their content, including one hypothetical and 18 known proteins, could be identified. Data are showed in Table 3 [53]–[70]. In relation to known proteins, the majority are commonly involved in the parasites' metabolism, such as nucleosome assembly proteins [57], 6-phosphogluconolactonase [62], and rieske iron sulfur proteins [66], while others, such as mannose-1-phosphate guanyltransferase [56] and short chain dehydrogenase [65], have been employed as candidates for immunotherapeutic targets.

**Table 2:** Identification of proteins that presented a significant decrease in their expression content.

					Mascot search results		Normalized values^h^							
Match ID^a^	Identification^b^	Uniprot ID^c^	*pI* (pred/exp)^d^	*Mr* (pred/exp)^e^	N^o^. match peptide^f^	Coverage (%)^g^	R0	R10	R20	R30	Fold^i^	One-way ANOVA (*P* value)^j^	Function^k^	Literature^l^
116	Hypothetical protein	E9AVJ0	4.65/5.63	12/55	4(1)	1	1.00	0.78	0.60	0.46	2.18	7,24E-04	Unknown	Unknown
141	Hypothetical protein	E9ANW9	4.90/4.84	15/18	2(2)	8	1.00	0.66	0.59	0.48	2.09	6,91E-03	Unknown	Unknown
142	Small myristoylated protein-3	E9APT0	4.51/4.70	15/13	6(4)	32	1.00	0.75	0.48	0.46	2.18	7,97E-07	Unknown	Infectivity [22]
169	Small GTP-binding protein Rab1	E9AYX8	5.36/5.54	19/22	12(8)	60	1.00	0.62	0.50	0.32	3.13	4,95E-03	Transport	Infectivity [23]
184	Peroxidoxin	E9AW04	6.27/6.90	20/26	2(2)	6	1.00	0.78	0.45	0.42	2.38	3,24E-03	Metabolism	Vaccine [24]
185	Tryparedoxin Peroxidase I	Q4QF76	6.19/6.79	20/25	13(10)	21	1.00	0.75	0.56	0.48	2.09	3,08E-03	Metabolism	Infectivity [25]
260	Succinyl-CoA ligase [GDP-forming] beta-chain,putative	E9AT73	5.44/6.77	28/45	6(4)	12	1.00	0.80	0.63	0.38	2.63	4,90E-03	Metabolism	Infectivity [26]
291	α-tubulin	E9AP62	5.33/5.45	31/61	24(22)	31	1.00	0.81	0.58	0.47	2.13	7,89E-06	Structural	Infectivity [27]
312	Aldehyde dehydrogenase	E9AXJ1	6.67/7.52	34/55	2(1)	11	1.00	0.62	0.43	0.43	2.33	2,24E-05	Metabolism	Metabolism [28]
336	Metallo-peptidase, Clan MA(E), Family M32	E9B493	5.26/5.51	36/57	19(8)	23	1.00	0.81	0.59	0.44	2.27	3,67E-06	Protein synthesis	Infectivity [29]
388	Paraflagellar rod protein 1D	E9ALP7	5.34/5.36	44/69	12(7)	16	1.00	0.89	0.62	0.37	2.71	1,52E-04	Structural	Infectivity [30]
412	Eukaryotic translation initiation factor 3 subunit 8	E9AUD5	4.05/5.64	48/82	4(3)	4	1.00	0.44	0.37	0.27	3.70	4,87E-03	Protein synthesis	Metabolism [31]
615	Hypothetical protein	E9ASM0	6.61/7.09	27/147	1(1)	0	1.00	0.88	0.63	0.37	2.71	9,05E-03	Unknown	Unknown
640	Actin	P45520	5.85/5.40	34/42	4(2)	13	1.00	0.51	0.40	0.18	5.56	6,49E-03	Structural	Metabolism [32]
646	Heat shock 70 kDa protein	Q07437	4.88/6.05	39/45	13(11)	14	1.00	0.78	0.40	0.37	2.71	1,56E-03	Protein folding	Infectivity [33]
653	Eukaryotic initiation factor 4A	O62591	4.65/5.83	42/45	8(4)	13	1.00	0.99	0.85	0.38	2.63	9,39E-03	Protein synthesis	Vaccine [34]
656	Paraflagellar rod protein 2C	E9AQV6	5.18/5.73	43/77	4(1)	5	1.00	0.66	0.37	0.43	2.71	1,57E-04	Structural	Infectivity [35]
697	Hypothetical protein	E9B489	5.27/5.36	25/36	1(1)	4	1.00	0.60	0.55	0.41	2.44	1,47E-03	Unknown	Unknown
776	Enolase	E9APW3	5.80/5.48	30/47	4(3)	13	1.00	0.58	0.45	0.36	2.78	9,87E-04	Metabolism	Infectivity [36]
69	Glutamine synthetase	E9AKR5	5.81/5.71	38/43	4(3)	12	1.00	0.82	0.50	0.47	2.13	2,29E-05	Metabolism	Vaccine [37]
76	Malic enzyme	E9AWR7	5.01/5.79	39/63	13(8)	18	1.00	0.81	0.32	0.27	3.70	2,85E-04	Metabolism	Vaccine [38]
77	Putative phosphatase 2C	E9B0G2	4.96/4.93	41/43	8(7)	20	1.00	0.51	0.38	0.33	3.03	1,22E-03	Cell signaling	Infectivity [39]
107	Elongation factor 2	E9ASD6	6.03/5.77	12/95	1(1)	1	1.00	0.53	0.58	0.48	2.08	5,56E-03	Protein synthesis	Vaccine [40]
125	Endoribonuclease L-PSP (pb5)	E9AW21	5.86/5.52	13/17	2(2)	9	1.00	0.72	0.45	0.40	2.50	1,98E-03	DNA binding protein	Therapeutic [41]
149	Ribonucleoprotein p18	E9AQ29	5.09/5.55	16/22	4(0)	15	1.00	0.55	0.44	0.50	2.27	2,12E-04	DNA binding protein	Metabolism [42]
210	Hypothetical protein	E9AXT3	5.64/5.64	23/31	13(9)	29	1.00	0.61	0.50	0.58	2.04	1,10E-04	Unknown	Unknown
211	Hypothetical protein	E9B549	4.37/9.90	23/23	5(5)	25	1.00	0.51	0.47	0.58	2.08	1,56E-03	Unknown	Unknown
235	Metallo-peptidase, Clan ME, Family M16	E9B2A8	6.64/5.06	25/120	1(1)	1	1.00	0.64	0.47	0.55	2.22	1,43E-04	Protein synthesis	Infectivity [43]
239	β-tubulin	E9AMJ8	4.61/5.95	25/47	20(15)	32	1.00	0.56	0.42	0.47	2.38	2,64E-05	Structural	Infectivity [44]
262	Chain A, Open And Closed Structures Of The Udp-Glucose Pyrophosphorylase From *Leishmania Major*	Q4QDU3	4.92/5.84	28/56	2(1)	2	1.00	0.65	0.49	0.42	2.38	8,46E-03	Metabolism	Metabolism [45]
296	Peptidase m20/m25/m40 family-like protein	E9B1Y8	5.04/5.10	32/38	5(5)	15	1.00	0.71	0.49	0.45	2.04	5,10E-04	Protein synthesis	Metabolism [46]
308	S-adenosylmethionine synthetase	E9B1C6	5.12/5.42	34/44	9(5)	16	1.00	0.91	0.58	0.49	2.04	3,36E-03	Metabolism	Metabolism [47]
381	Protein disulfide isomerase	E9AUD1	5.06/5.04	42/53	12(7)	22	1.00	0.90	0.44	0.41	2.44	1,05E-03	Metabolism	Infectivity [48]
519	Eukaryotic translation initiation factor 3 subunit	E9ATH0	5.14/5.21	35/39	7(5)	14	1.00	0.42	0.32	0.22	4.55	2,13E-10	Protein synthesis	Metabolism [49]
584	Basic transcription factor 3a	E9ATF9	4.00/9.44	13/12	1(1)	15	1.00	0.37	0.24	0.26	3.85	6,69E-04	Protein synthesis	Metabolism [50]
586	60S acidic ribosomal protein P2-2	Q06382	4.04/4.23	13/11	8(8)	52	1.00	0.35	0.34	0.26	3.85	2,51E-03	Protein synthesis	Infectivity [51]
606	Thiol-dependent reductase 1	E9B3K3	6.38/5.65	24/46	3(2)	12	1.00	0.38	0.27	0.22	4.55	1,71E-05	Unknown	Vaccine [52]

a ^)^ Spots match ID number obtained from ImageMaster Platinum;

b ^)^ Name of the identified protein;

c ^)^ Uniprot identification code;

d ^)^ Experimentally predicted and expected isoelectric point (*pI*);

e ^)^ Experimentally predicted and expected molecular weight (*Mr*, in kDa);

f ^)^ Number of identified peptides by MS;

g ^)^ Percentage of the protein sequence covered by identified peptides;

h ^)^ Normalized data from R0 represented by mean values of each condition divided by R30 value;

i ^)^ Fold represents the maximum spot intensity mean value of the conditions divided by the smallest value;

j ^)^ One-way ANOVA (*P*<0.01) obtained from spot analysis;

k ^)^ Biological functions according to NCBI, UniProt, and Gene Ontology databases;

l ^)^ Biological activity and/or immunological application described in other studies: [22] Tull et al., 2010; [23] Oliveira et al., 2006; [24] Daifalla et al., 2011; [25] Iyer et al., 2008; [26] Hunger-Glaser et al., 1999; [27] Werbovetz et al., 1999; [28] Feng et al., 2011; [29] Niemirowicz et al., 2007; [30] Hunger-Glaser et al., 1997; [31] Alcolea et al., 2009; [32] Bhaskar et al., 2012; [33] Khanra et al., 2012; [34] Berberich et al., 2003; [35] Moore et al., 1996; [36] Swenerton et al., 2011; [37] Hummadi et al., 2006; [38] Martins et al., 2006; [39] Burns et al., 1993; [40] Kushawaha et al., 2011; [41] Misra et al., 2005; [42] Bringaud et al., 1995; [43] Eggleson et al., 1999; [44] Mureev et al., 2007; [45] Steiner et al., 2007; [46] Martínez-Rodríguez et al., 2012; [47] Drummelsmith et al., 2004; [48] Achour et al., 2002; [49] Buda et al., 2013; [50] Alcolea et al., 2011; [51] Martín et al., 2009; [52] Silva et al., 2012. The proteins were identified through the data included in the NCBI database (dated June 2012) for *Leishmania spp*.

**Table 3:** Identification of proteins that presented a significant increase in their expression content.

					Mascot search results		Normalized values^h^							
Match ID^a^	Identification^b^	Uniprot ID^c^	*pI* (pred/exp)^d^	*Mr* (pred/exp)^e^	N^o^. match peptide^f^	Coverage (%)^g^	R0	R10	R20	R30	Fold^i^	One-way ANOVA (*P* value)^j^	Function^k^	Literature^l^
8	Calreticulin	E9B259	4.52/4.51	50/45	3(3)	5	1.00	3.26	4.40	4.49	4.49	6.96E-03	Protein folding	Metabolism [53]
12	Isocitrate dehydrogenase	E9B494	5.44/5.51	40/47	16(6)	28	1.00	1.71	1.76	2.51	2.51	2.41E-03	Metabolism	Metabolism [54]
303	60S acidic ribosomal subunit protein	E8NHJ8	5.07/5.00	33/35	26(21)	45	1.00	3.15	4.72	4.75	4.75	9.76E-05	Protein synthesis	Diagnosis [55]
326	Mannose-1-phosphate guanyltransferase	E9AW11	5.67/5.29	36/42	10(7)	23	1.00	2.07	2.25	3.24	3.24	1.72E-04	Metabolism	Metabolism [56]
392	Nucleosome assembly protein	E9ARZ6	4.64/4.64	45/40	17(9)	25	1.00	2.17	2.49	2.61	2.61	1.78E-04	DNA binding protein	Metabolism [57]
420	ATPase beta subunit	E9AXJ6	5.02/5.14	49/56	60(51)	49	1.00	1.74	1.89	2.02	2.02	9.55E-04	Metabolism	Metabolism [58]
432	T-complex protein 1, theta subunit	E9AUC7	5.27/5.24	54/59	27(18)	49	1.00	1.54	2.05	3.35	3.35	3.65E-04	Protein folding	Metabolism [59]
458	Chain A, Protein Structure Of Usp From *L. Major* in Apo-Form	D3G6S4	5.36/5.34	63/69	4(4)	3	1.00	1.82	3.11	3.24	3.24	2.57E-03	Metabolism	Metabolism [60]
739	Hs1vu complex proteolytic subunit-like,hs1vu complex proteolytic subunit-like, threonine peptidase, Clan T(1), family T1B	E9ATI1	5.24/6.09	22/25	4(1)	9	1.00	1.65	1.87	2.05	2.05	3.70E-04	Protein synthesis	Metabolism [61]
767	6-phosphogluconolactonase	E9AYQ1	5.50/5.22	26/29	2(2)	8	1.00	1.39	1.71	2.27	2.27	1.63E-03	Metabolism	Metabolism [62]
40	Heat shock protein 83; HSP 83	P27741	6.27/5.00	31/81	1(1)	1	1.00	1.88	2.92	2.93	2.93	3.76E-03	Protein folding	Diagnosis [63]
62	2-hydroxy-3-oxopropionate reductase	E9B0E2	5.77/5.40	26/31	6(5)	25	1.00	2.39	3.13	3.42	3.42	6.53E-05	Metabolism	Metabolism [64]
230	Short chain dehydrogenase	E9B602	6.57/6.31	25/28	3(1)	9	1.00	2.16	2.61	2.62	2.62	8.48E-04	Metabolism	Therapeutic [65]
279	Reiske iron-sulfur protein precursor	E9B632	5.57/6.02	29/34	9(7)	43	1.00	1.83	2.43	2.89	2.89	6.34E-06	Metabolism	Metabolism [66]
327	Vacuolar ATPase subunit-like protein	E9AKM1	4.93/4.85	36/42	13(5)	25	1.00	1.75	2.39	2.49	2.49	3.74E-03	Metabolism	Metabolism [67]
510	Cyclin 1	E9AMR1	5.99/5.67	31/36	3(1)	13	1.00	2.26	2.28	2.78	2.78	8.78E-03	Protein synthesis	Metabolism [68]
529	Protein transport protein Sec13	E9B2C5	5.69/5.51	34/37	2(1)	9	1.00	2.45	2.78	3.00	3.00	2.79E-03	Unknown	Metabolism [69]
676	Hypothetical protein	E9ATK7	4.98/4.91	98/119	33(22)	27	1.00	1.75	2.75	2.80	2.80	3.84E-03	Unknown	Unknown
735	Glucose-regulated protein 78; GRP78	E9AZT9	5.15/5.18	67/72	27(22)	28	1.00	4.69	4.84	4.87	4.87	9.57E-05	Protein folding	Vaccine [70]

a ^)^ Spots match ID number obtained from ImageMaster Platinum;

b ^)^ Name of the identified protein;

c ^)^ Uniprot identification code;

d ^)^ Experimentally predicted and expected isoelectric point (*pI*);

e ^)^ Experimentally predicted and expected molecular weight (*Mr*, in kDa);

f ^)^ Number of identified peptides by MS;

g ^)^ Percentage of the protein sequence covered by identified peptides;

h ^)^ Normalized data from R0 represented by mean values of each condition divided by R30 value;

i ^)^ Fold represents the maximum spot intensity mean value of the conditions divided by the smallest value;

j ) One-way ANOVA (*P*<0.01) obtained from spot analysis;

k ^)^ Biological functions according to NCBI, UniProt, and Gene Ontology databases;

l ^)^ Biological activity and/or immunological application described in other studies: [53] Joshi et al., 1996; [54] Tielens et al., 2010; [55] Soto et al., 1996; [56] Lackovic et al., 2010; [57] Scher et al., 2012; [58] Sánchez-Cañete et al., 2009; [59] Peris et al., 1994; [60] Steiner et al., 2007; [61] Jaramillo et al., 2011; [62] Duclert-Savatier et al., 2009; [63] Celeste et al., 2004; [64] Liu et al., 2011; [65] Leblanc et al., 1998; [66] Priest et al., 1996; [67] Bakker-Grunwald, 1992; [68] Banerjee et al., 2006; [69] Casanova et al., 2008; [70] Jensen et al., 2001. The proteins were identified through the data included in the NCBI database (dated June 2012) for *Leishmania spp.*

### Immunoblotting validation

**Figure 3:**
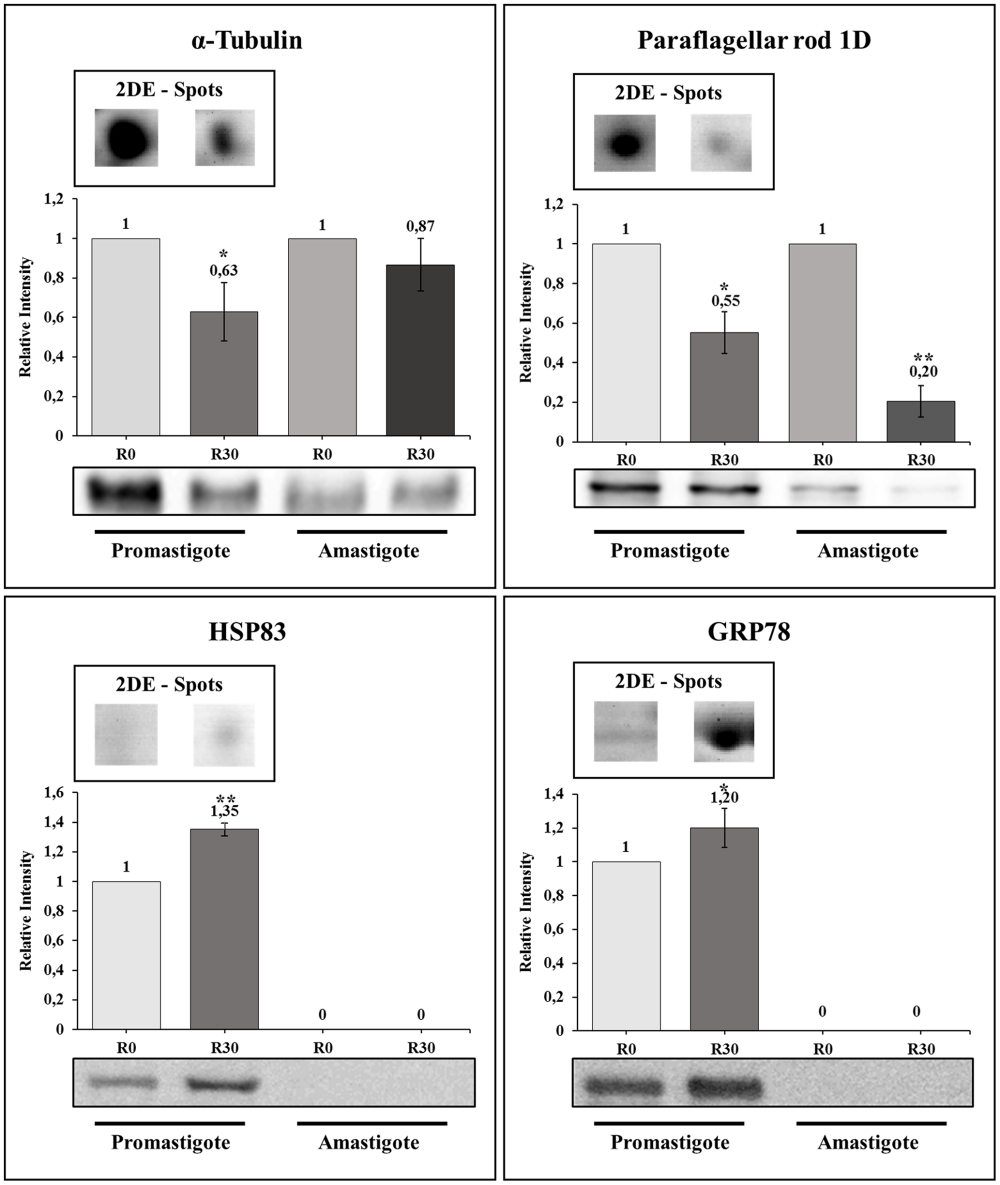
Immunoblotting validation of some proteins in *Leishmania amazonensis*. Representative immunoblotting of some proteins that presented a significant decrease or increase in their expression content between R0 and R30 passages, using promastigote and amastigotes-like forms of *L. amazonensis*, are shown here. For each protein [α-tubulin, in A; paraflagellar rod protein 1D, in B; glucose-regulated protein 78 (GRP78) in C, and heat shock protein 83 (HSP83), in D], this image presents one example of correspondent 2-DE spot of promastigote form obtained from R0 or R30 passages. The antibodies used to validate each spot are described in the material and methods section. Asterisks represent the comparison between the expression of the protein in the R0 condition in relation to the R30 sample in each parasite stage, applying the Student's t-test (*P*<0.05), and the numbers represent the relative variation of each protein in comparison to R0 of each parasite stage. All experiments were performed in triplicate.

Some of the identified proteins that presented a significant increase or decrease in their contents in the axenic cultures were used to validate the results found in this study. In this context, two of them presenting a significant decrease in their expression content, namely α-tubulin and paraflagellar rod protein 1D, and two of them, which presented an increase in their expression, namely HSP83 and GRP78, were used in the Western blot experiments (Figure 3). When promastigote extracts were employed, the selected proteins showed a variation that runs in line with the results obtained in the 2-DE gels. In addition, the decrease in the level of α-tubulin and paraflagellar rod protein 1D detected in the promastigote forms are also maintained when the R30 forms are axenically derived into the amastigote stage of the parasite.

## Discussion

*Leishmania amazonensis* is a member of the *Leishmania mexicana* complex, and it is the etiological agent for a broad spectrum of disease in South American countries [5]. The mechanisms of *in vivo* persistence are of particular interest to this parasite species, given that several lines indicate that *L. amazonensis*, when compared to other *Leishmania* species, is particularly adept at surviving attacks from intracellular killing mechanisms [5], [14]. Taking this into account, the present study applied a proteomic approach to analyze the variation of the protein expression profile from *L. amazonensis*, which was pre-isolated from lesions of chronically infected BALB/c mice and maintained in *in vitro* cultures over a long period of time. The purpose of this study was to verify whether or not the *in vitro* cultivation, performed over a 150-day period, could in fact decrease the parasites' infectivity, as well as to identify proteins that could present a relation with a possible loss of infectivity in *L. amazonensis*.

Studies have shown that the maintenance of *Leishmania* in axenic cultures over long periods of time constitutes a relevant factor in the reduction of infectivity in *L. infantum* [71] and *L. major* [72]. In one study, the loss of infectivity in *L. infantum* was related to the maintenance of the parasites after 105 days of successive *in vitro* passages [73]. Proteomic analyses have been employed successfully to identify proteins expressed in both promastigote and amastigote stages of *Leishmania spp.*, as well as to evaluate the stage-specific proteins and protein expression profile in the parasites [8], [11], [74], [75], [76], [77]. In the present study, proteins that presented a significant variation in their content, observed using 2-DE gels and analyzed by bioinformatics programs, were identified in an attempt to select possible targets for future immunological interventions in leishmaniasis. For this, stationary promastigotes were used in the same concentration in all passages so as to perform the experiments properly. In general, an increase of *Leishmania* promastigote infectivity can also be observed when parasites pass from the logarithmic phase (days 1–3) to the stationary phase (days 4–6) of their growth cycle in *in vitro* cultures [78], [79], [80], [81]. In the present study, it could be observed that the percentage of the stationary promastigotes found in all cultures was homogeneous, suggesting that the changes found in the protein expression profile and in the infectivity values of the parasites submitted to axenic cultures, could not only be associated with or depend on the reduction in the number of infective promastigotes present in the *in vitro* cultures.

Another important aspect here was the reduction in the *in vitro* and *in vivo* infectivity observed from R0 to R30 samples. In the *in vitro* experiments performed using murine macrophages, in addition to a significant decrease found in the percentage of infected macrophages, a marked reduction in the number of intracellular amastigotes could be observed. Evaluating *in vitro* cultures performed up to 300 days after infection (R60), as compared to R30, no significant difference was found in the percentage of infected macrophages, and in the number of intra-macrophage amastigotes (data not shown). In addition, when R0 and R30 cultures were used to infect BALB/c mice, it could be observed that animals infected with R0 developed a more progressive disease than did those infected with the R30 sample, confirming the results obtained from *in vitro* experiments, though no significant difference could be observed between R20 and R30 in the infectivity experiments. Furthermore, the present study's data are in accordance with Moreira et al. (2012), which showed that *L. infantum* promastigotes present a significant loss of their infectivity after 100 days of *in vitro* cultures, suggesting that this condition may well be related to specific modifications in the protein differentiation content of parasites [73].

In relation to the identified proteins that presented a decreased expression from R0 to R30, several had already been described in other published studies, such as proteins involved in the infectivity of *Leishmania* or in other parasite species. For example, peroxidoxin is a protein expressed in the endoplasmic reticulum of Trypanosomatides and is involved in cellular resistance to reactive oxygen species [82], been also a virulence factor described in *Trypanosoma cruzi* [83]. The malic enzyme is involved in the virulence of *Xanthomonas campestris* [84], while aldehyde dehydrogenase acts in the protection of mammal cells against damage evoked by osmotic and saline stress [85]. S-adenosylmethionine synthetase in *L. panamensis* [86] and *L. major* [87] is related to drug resistance. Enolase is a membrane protein that plays a role in the infectivity of *Leishmania*, as it is involved in the interaction between the parasites and host cells [88]. The carboxypeptidase family (M32) has also been identified as a virulence factor in *T. cruzi* [89] and operates in the catabolism of peptides, favoring the growth and multiplication of parasites [90]. Phosphatase 2C is considered a virulence factor in *Toxoplasma gondii* [91], while tryparedoxin peroxidase in *L. donovani* is involved in drug resistance [92].

Evaluating the databases of proteins that presented an increased expression from R0 to R30, most present metabolic functions described in prior literature, such as those related to cellular stress, recovery of improperly folded proteins, and the restoration of core functions. In this context, phosphatase 1 guanyltransferase mannose is involved in oxidative stress in yeast [93], while isocitrate dehydrogenase is involved in cellular stress in *Cryptococcus neoformans* [94]. The glucose regulated protein 78 kDa is a membrane protein that is up-regulated in conditions of cellular stress and that can lead to cell cycle arrest [95]. The protein complex Hs1VU-like proteolytic subunit is a peptidase that is over-expressed and correlated to the accumulation of improperly folded proteins within the cells [96]. Calreticulin is involved in cellular processes related to protein folding, calcium homeostasis, apoptosis, and cell differentiation [97].

Western blot assays with four identified proteins were performed to validate 2-DE gel quantification results. When promastigote samples were analyzed, a significant correlation could be observed when comparing the two techniques used for proteins with a decreased or increased expression in aged cultures (Figure 3). When axenic amastigote extracts were employed for Western blots, a decrease in the level of α-tubulin and paraflagellar rod protein 1D observed in the 2-DE was also detected. Unfortunately, the lack of signs when antibodies against HSP83 and GRP78 were employed made it impossible to confirm whether or not the increase in protein expression associated with the loss of infectivity is maintained in the amastigote forms.

In conclusion, the data presented in the present study could contribute to a better understanding of the biological processes involved in a possible loss of infectivity of *L. amazonensis* when submitted to *in vitro* cultures over a long period of time, as described for other *Leishmania* species. Furthermore, the identified proteins presenting a significant decrease in their protein content during cultivation, including the hypothetical, should be evaluated in future studies, including vaccine candidates and/or immunotherapeutic targets against leishmaniasis. Additional studies are warranted in an attempt to address the major concern that identified proteins are indeed involved in the possible loss of virulence in the parasites cultured over long periods of time.
