# Correction: Identification of Differentially Expressed Proteins from *Leishmania amazonensis* Associated with the Loss of Virulence of the Parasites

**DOI:** 10.1371/journal.pntd.0002956

**Published:** 2014-05-22

**Authors:** The PLOS Neglected Tropical Diseases Staff

The thirteenth author’s name is spelled incorrectly. The correct name is: Ronaldo A. P. Nagem.
